# Dual-targeted nano-encapsulation of neonatal porcine islet-like cell clusters with triiodothyronine-loaded bifunctional polymersomes

**DOI:** 10.1186/s11671-024-03964-3

**Published:** 2024-02-05

**Authors:** Sang Hoon Lee, Minse Kim, Eun-Jin Lee, Sun Mi Ahn, Yu-Rim Ahn, Jaewon Choi, Jung-Taek Kang, Hyun-Ouk Kim

**Affiliations:** 1 MGENSolutions Biotechnology Research Institute, Seoul, 06688 Republic of Korea; 2 Department of Biotechnology and Bioengineering, Kangwon National University, Chuncheon, Gangwon-do 24341 Republic of Korea; 3 Biohealth-Machinery Convergence Engineering, Kangwon National University, Chuncheon, Gangwon-do 24341 Republic of Korea

**Keywords:** Drug carrier, Dual targeting, Nano-encapsulation, Neonatal porcine islet-like cell clusters (NPCCs), Polymersomes

## Abstract

There is growing evidence that neonatal porcine islet-like cell clusters (NPCCs) isolated from piglets can be used to treat type 1 diabetes in humans. However, graft rejection is a common complication in humans owing to the prevalence of xenoantigens in porcine. Therefore, researchers have investigated various islet encapsulation techniques that could protect against these antigens. To this end, this study presents a robust nano-encapsulation method based on bifunctional polymersomes (PSomes), in which N-hydroxysuccinimide (NHS) and maleimide (Mal) groups conjugated to the PSomes terminal interact with the amine and thiol groups on the surface of NPCCs to induce dual targeting via two covalent bonds. The findings indicate that the ratio of NHS to Mal on PSomes is optimal for dual targeting. Moreover, triiodothyronine (T3) is known to promotes pancreatic islet maturation and differentiation of endocrine cells into beta cells. T3 encapsulated in PSomes is shown to increase the glucose sensitivity of NPCCs and enhance insulin secretion from NPCCs. Furthermore, improvements in the nano-encapsulation efficiency and insulin-secreting capability of NPCCs through dual targeting via dual-Psomes are demonstrated. In conclusion, the proposed nano-encapsulation technique could pave the way for significant advances in islet nano-encapsulation and the imprevement of NPCC immaturity via T3 release.

**Supplementary Information:**

The online version contains supplementary material available at 10.1186/s11671-024-03964-3.

## Introduction

Type 1 diabetes (T1D) is characterized by the destruction of insulin-producing beta cells in the pancreas that results in a chronic autoimmune disease. The symptoms of T1D include immune dysfunction, elevated blood glucose levels, and numerous other complications [1, 2]. According to a study published by the International Diabetes Federation, the incidence of T1D is increasing worldwide [3]. Although pancreatic islet transplantation has the potential to be an effective treatment for T1D, the lack of suitable donors is a significant obstacle preventing the full realization of standard treatment options [4]. Using porcine islets for transplantation has been investigated as a potentially advantageous alternative for avoiding this obstacle.

Neonatal porcine islet-like cell clusters (NPCCs) have been a feasible transplantation option for the last few years [5]. NPCCs offer several advantages that distinguish them from adult porcine islets (APIs), including low cost, simple accessibility, standardized isolation processes, and hypoxic resistance [6, 7]. However, the transplantation of pig islets into humans or nonhuman primates has proven problematic because of the risk of severe rejection caused by the pre-existing natural antibodies that activate an early immune response in the blood, known as the instant blood-mediated inflammatory reaction [8]. As a result, the transplantation of porcine islets into humans has become an exceedingly difficult task. Therefore, to solve the problem, various methods for islet encapsulation have been developed as a consequence of previous research that employed biocompatible materials to circumvent the immunological barrier.

Nano-encapsulation is a relatively novel technique that has emerged as a potentially effective method for enhancing the function of islets while shielding them from attacks by the immune system [9]. This method facilitates the efficient transport of essential components, such as oxygen, nutrients, and hormones, regardless of islet sizes, also allows for a relatively small transplantation volume [10]. Polymeric vesicles, also known as polymersomes (PSomes), are one of the many materials that can be used for nano-encapsulation [11]. They have attracted the most attention because of their potential use in islet xenotransplantation. PSomes are spherical amphiphilic vesicles that are predominantly composed of polyethylene glycol (PEG) [12]. Their structure consists of a hydrophilic core and a hydrophobic shell. One unexpected advantage of this one-of-a-kind structure is that it can suppress the immune system [13]. The ability of PSomes to transfer hydrophilic or hydrophobic drugs into their core or shell, thereby producing a stable and prolonged drug release pattern, is an additional benefit of these delivery systems [14]. Islet encapsulation commonly involves the formation of a covalent bond between an amine group on the islet surface and N-hydroxysuccinimide (NHS)-conjugated substances. This was performed to preserve islet integrity [15–17]. The thiol–maleimide (Mal) reaction is an additional form of binding that can occur in a neutral pH environment [18]. This reaction requires a thiol group on the cell membrane or extracellular matrix (ECM) protein [19].

NPCCs do not produce sufficient insulin in response to variations in glucose concentration because they lack the same level of functional maturity as adult porcine islets. Consequently, NPCC-based therapies require significantly more time to maintain stable blood glucose levels after transplantation [20]. Numerous studies have attempted to overcome this limitation by enhancing the insulin-secreting capacity of the NPCCs. Triiodothyronine (T3) is a thyroid hormone that has shown promise in promoting the maturation of endocrine cells into insulin-secreting beta cells and in enhancing the functionality of pancreatic islets [21, 22]. Moreover, T3 promotes the transdifferentiation of pancreatic ductal cells into beta cells, resulting in an increase in the insulin-secreting capacity of NPCCs [22].

**Scheme 1:**
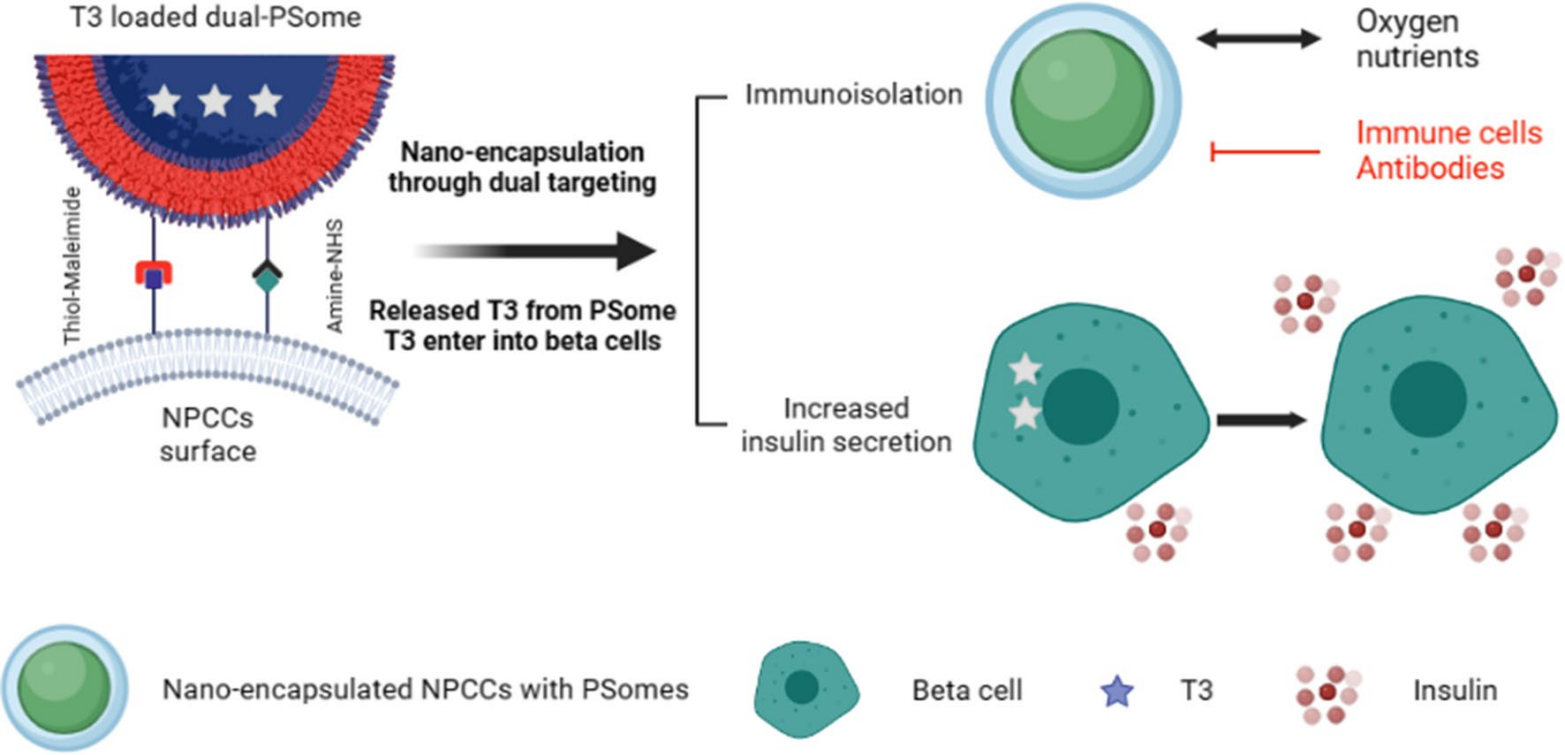
Encapsulation is intended for both immunoisolation and insulin secretion enhancement. Owing to their immaturity, NPCCs are incapable of regulating hyperglycemia. When T3 is incorporated into dual-PSomes, it is released and incorporated into the NPCC’s beta cells. Consequently, NPCCs encapsulated with T3-loaded dual-PSomes acquire protection against the host's immune response and manifest an insulin secretion potential further enhanced by T3

In this study, we designed a nanoplatform for dual targeting in nano-encapsulation by coupling NHS and maleimide to form bifunctional PSomes (dual-PSomes). This technique permits the covalent attachment of amine and thiol groups to the surfaces of NPCCs. By increasing the efficacy of encapsulation, we aimed to develop a robust immunoisolation barrier around the NPCCs, thereby protecting them from potential immune attacks. In addition, in this study, we examined the viability of T3-loaded dual PSomes encapsulation and enhanced the insulin-secreting capability of NPCCs, with the responsiveness of NPCCs determined by the glucose concentration in the surrounding environment (Scheme 1). This research has the potential to lead to the development of nano-encapsulation methods and a novel strategy for NPCCs maturation through dual targeting and prolonged T3 release, which can improve the treatment for T1D. Our results will provide useful insights into the field of islet xenotransplantation by addressing the limitations of NPCCs and improving their capabilities.

## Materials and methods

### Materials

NHS-PEG-b-PLA copolymers and Mal-PEG-b-PLA copolymers with a molecular weight of 6000 Da were purchased from Nanosoft Polymers. DiI was purchased from Biotium. Ham’s F10 medium and bovine serum albumin (BSA) were obtained from Gibco and GenDEPOT, respectively. Dulbecco’s modified Eagle’s medium (DMEM), fetal bovine serum (FBS), and antibiotic-antimycotics were purchased from Biowest. The DNA Mini Kit and RNeasy Mini Kit were purchased from QIAGEN. TOPscript™ complementary DNA (cDNA) Synthesis Kit and qPCR SyGreen Mix were purchased from Enzynomics and PCR BIOSYSTEMS, respectively. SnakeSkin™ Dialysis Tubing (10,000 MWCO) was purchased from Thermo and pluriStrainer® 500 µm was purchased from pluriSelect. 3-(4,5-Dimethylthiazol-2-yl)-2,5-diphenyltetrazolium bromide (MTT) cell proliferation assay kit was obtained from iNtRON Biotechnology. Hank’s balanced salt solution (HBSS), dimethyl sulfoxide (DMSO), nicotinamide, D-glucose, L-glutamine, calcium chloride dihydrate, isobutylmethylxanthine, ciprofloxacin, N-(2-Hydroxyethyl) piperazine-N′-(2-ethanesulfonic acid) (HEPES), sodium bicarbonate, collagenase type V, acridine orange (AO), propidium iodide (PI), and 3,3',5-triiodo-L-thyronine sodium salt (T3) were purchased from Sigma Aldrich. Human/Canine/Porcine Insulin Quantikine ELISA Kit purchased from R&D Systems. All other chemicals and reagents were of analytical grade.

## Animals

The experimental protocols involving animals were approved by the Institutional Animal Care and Use Committee of the Institute of MGENSolutions Co. Ltd. (approval number: #2019–1). All procedures were performed in accordance with the committee's stipulations. The piglets were anesthetized using general anesthesia. Careful measures were taken to minimize discomfort during the procedure. Pancreatectomy was performed on the piglets as part of the study protocol. Prior to the surgical excision of the pancreas, all piglets were euthanized to underscore humane treatment.

## Production of PSomes

PEG-b-PLA copolymer conjugates (PSomes, 5 mg/mL) with NHS- and Mal- groups were dissolved in 1 mL of DMSO to prepare PSomes. Dual-PSomes containing both NHS- and Mal- were formed by combining individual copolymers in varying proportions. To this solution, 3 mg of T3 and DiI were added for drug loading and visualization, respectively. After adding 4 mL of distilled water, the solution was ultrasonicated at 60 °C for 10 min to obtain a final concentration of 1 mg/mL. The resulting mixture was dialyzed for 3 days using SnakeSkinTM Dialysis Tubing (10,000 MWCO) in distilled water.

## Characterization of PSomes

At 25 °C, the hydrodynamic dimension of PSomes was determined using dynamic dynamic light scattering (DLS) with ELSZ-2000S (Otsuka Electronics Co). Simultaneously, their zeta potentials were measured using a Zetasizer Nano ZSP (Malvern Panalytical). The morphology of PSomes was examined using JEM-2100F (Jeol Ltd.) field emission Transmission Electron Microscope (TEM) at an accelerating voltage of 2000 kV. For the TEM analysis, 10 μl of PSome dispersion was applied to formvar/carbon-coated 75-mesh copper grids (Ted Pella), negatively stained with a 1% (w/v) of uranyl acetate aqueous neutral solution, and air-dried for 30 min. Additionally, PSomes stability was measured for up to 8 months using DLS.

## Analysis of T3 release in vitro

Release of T3 contained in Dual-PSomes was performed using 0.01 M NaOH and Tube-O-DIALYZER (Medi 50 K MWCO, GenoTechnology Inc., St Louis, MO, USA). The amount of T3 was determined by analyzing the drug content using High-performance liquid chromatography (HPLC). Furthermore, the in vitro T3 release pattern was measured using HPLC. The analysis was conducted using liquid chromatography (AGILENT 1260 Infinity, Waldbronn, Germany) equipped with a diode array detector and Quaternary Gradient pump, and separation and quantification were performed on an Eclipse XDB-C18 4.6 × 250, 5-µ column. The column was maintained at 4 °C, and the flow rate and injection volume were 1 mL/min and 20 µL, respectively. The mobile phase was a mixture of methanol/deionized water (65:35, v/v) with 2% acetic acid. The detection was performed at a wavelength of 240 nm. As a result, T3 was released from T3 loaded NHS:Mal (7:3)-dual-PSomes for up to 2 weeks.

## Isolation of neonatal porcine islet-like cell clusters (NPCCs)

NPCCs were extracted from the pancreata of piglets aged 3–5 days via enzymatic digestion. The procedure for isolating NPCCs has been previously described.^15^ Briefly, following the humane sacrifice of piglets, the pancreas was carefully dissected to prevent a bowel injury. The pancreas was immediately homogenized into 1–2-mm^3^-sized fragments. Collagenase type V (2.5 mg/mL) was added to HBSS containing 8.3 mM of sodium bicarbonate, 10 mM of HEPES, and 0.5% of antibiotic–antimycotic. Introducing an HBSS buffer containing 10% FBS halted the enzymatic activity. After two cycles of HBSS buffer rinsing, tissue samples were passed through a cell strainer with 500-μm pores. Postisolation, the NPCCs were incubated in Ham's F10 medium enriched with 0.25% BSA, 10 mM of nicotinamide, 10 mM of D-glucose, 2 mM of L-glutamine, 2 mM of calcium chloride dihydrate, 50 μM of isobutylmethylxanthine, 20 μg/mL of ciprofloxacin, and 1% of antibiotic–antimycotic. The cultivation conditions were maintained at 37 °C and 5% of CO_2_. NPCCs were cultured for 5–6 days, and the culture medium was replaced on the first and third postisolation days.

## Quality control of NPCCs

After culture, the number of NPCCs was determined by measuring the diameter of islet colonies with an ocular optical reticle. The calculated amount of NPCCs was denoted as the islet equivalent (IEQ). Using 0.67 µM of AO and 75 µM of PI, the viability of NPCCs was determined. The resulting viability data were normalized relative to the NTC, which served as the baseline.

## Glucose-stimulated insulin secretion assay

NPCCs were initially preincubated in Krebs–Ringer bicarbonate buffer (KRBB) enriched with 25 mM of HEPES, 115 mM of sodium chloride, 24 mM of sodium bicarbonate, 5 mM of potassium chloride, 1 mM of magnesium chloride hexahydrate, 2.5 mM of calcium chloride dihydrate, and 0.1% of BSA at a basal-glucose concentration of 2.8 mM for 1 h. NPCCs were then subjected to a sequential glucose challenge, in which they were exposed to low glucose levels (2.8 mM) in KRBB for 1 h, followed by a high glucose concentration (28 mM) in KRBB for another hour. Using the Human/Canine/Porcine Insulin Quantikine ELISA Kit, insulin secretion at both low and high glucose concentrations was evaluated by collecting the supernatants following incubation. To account for variations in cell numbers during insulin quantification, DNA was extracted from NPCCs participating in GSIS using a DNA preparation kit. Finally, insulin secretion levels under each condition were normalized relative to the NTC, which served as the baseline.

## Quantitative real-time polymerase chain reaction

RNA was obtained using an RNA prep kit. The extracted RNA was converted into cDNA using a cDNA synthesis reagent. qRT-PCR was performed using the qPCR SyGreen Mix to quantify the gene expression. This procedure was performed using Bio-Rad 's CFX Opus 96 Real-Time PCR System (Hercules, CA, USA). Forward—5'-CCAGCATCTGTTCCCTCTACC-3' and Reverse—5'-TTATTGGGTTTTGGGGTGCGG-3' were the specific primers designed for the porcine insulin gene. To assure the accuracy of gene expression data, the insulin gene's expression levels were referenced against GAPDH and then calculated using the _ΔΔ_Ct method.

## Nano-encapsulation

In 2.7 mL of ordinary F-10 medium supplemented with 0.25% BSA, approximately 10,000 IEQ NPCCs were plated. After the NPCCs were seeded, 300 µL of PSomes was added and allowed to interact with them for 1 h. Following treatment, the nano-encapsulated NPCCs were rigorously rinsed with HBSS and maintained in the F-10 culture medium. DiI-conjugated PSomes were visualized to facilitate fluorescence intensity–based observations.

## Efficiency of nano-encapsulation

NPCCs encapsulated with DiI-conjugated PSomes were visualized using a fluorescence microscope (Leica, Wetzlar, Germany), and detailed imaging was performed using CLSM from Carl Zeiss (Oberkochen, Germany). Red fluorescence emitted by PSomes conjugated with DiI and affixed to NPCCs was measured. This measurement was based on the MFI and was analyzed using ImageJ software provided by the National Institutes of Health (Bethesda, United States).

## Selective permeability assay

Dual-PSome–encapsulated NPCCs were subjected to a 2-h treatment with FITC-conjugated dextran of varying molecular weights (10, 20, 70, and 250 kDa). The internalization of FITC-labeled dextran within PSome nano-encapsulated NPCCs was determined using CLSM.

## MTT assay

HeLa cells were cultured in DMEM supplemented with 10% FBS and 1% penicillin–streptomycin at a density of 2 × 10^4^ cells per well in a 96-well plate. After 24 h, 20 µL of PSomes was added to 180 µL of DMEM, and the cells were incubated for an additional hour. The cells were then cleansed and grown in an appropriate culture medium. After an additional day, the MTT cell proliferation assay was performed to evaluate cell viability. The cell viability results were normalized to those of the NTC.

## Statistical analysis and graphing

Statistical analysis was conducted using GraphPad Prism 6.0 using the one-way (or two-way) analysis of variance. Statistical significance was denoted by * and **, corresponding to the P-values of 0.05 and 0.01, respectively. In addition, all graphs drawn in this study were generated using SigmaPlot 10.0 (Systat Software, Inc.).

## Results and discussion

### Characteristics of polymersomes

**Fig. 1:**
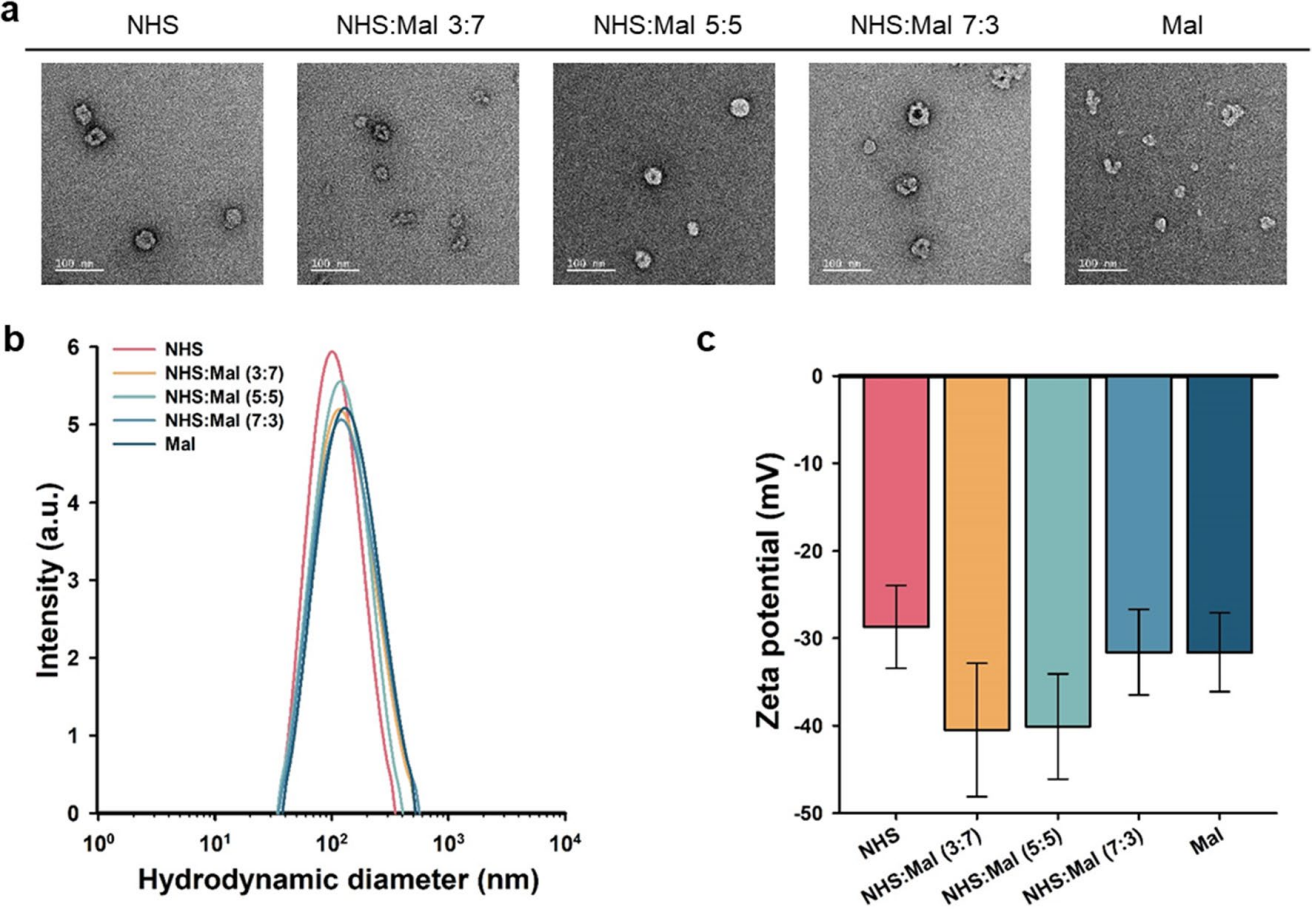
Characteristics of PSomes. **a** TEM images showcasing the morphological characteristics. Scale bars are set at 100 μm. **b** Size distribution profiles of PSomes, as determined using DLS. **c** Graph illustrating the zeta potentials of PSomes, indicating their surface charge (n = 3)

Amphiphilic self-assemblies, also known as polymersomes (Psomes), were created using PEG-block-poly lactide (PLA; PEG-b-PLA) copolymers as building blocks. Subsequently, the developed PSomes were examined and validated using various methods. When the morphology of PSomes was analyzed using transmission electron microscopy (TEM), all PSomes were found to have the homogeneous spherical form (Fig. 1a). This was the case regardless of the ratio of the functional groups in PSomes. Dynamic light scattering (DLS) was performed to determine the diameter of PSomes. As a consequence of these measurements, we discovered that the diameters of the NHS-, dual- (with NHS:Mal ratios of 3:7, 5:5, and 7:3), and Mal-PSomes were 114.8 (± 3.9), 149.9 (± 3.1), 129.3 (± 3.9), 155.2 (± 1.7), and 150.4 (± 4.8) nm, respectively (Fig. 1b). This result means that the polymerosomes we formed have diameters within the normal range of 50 to 250 nm, which is a normal diameter [12]. The zeta potentials of NHS-, dual- (with NHS:Mal ratios of 3:7, 5:5, and 7:3), and Mal-PSomes were -28.7 (± 4.73), − 40.5 (± 7.7), − 40.1 (± 6.0), − 31.6 (± 4.9), and − 31.6 (± 4.5) mV, respectively (Fig. 1c). All poroduced PSomes have conventional shapes, diameters, and charge characteristics. This suggests that the functional groups linked to PSomes may have the ability to bind to the NPCCs. This attachment might be facilitated by covalent bonds, which use the natural molecular characteristics of the functional groups. Finally, the representative dual-PSome (NHS:Mal 7:3) maintained its structural integrity for up to 8 months (Details in Supplement Fig. 2).

**Fig. 2:**
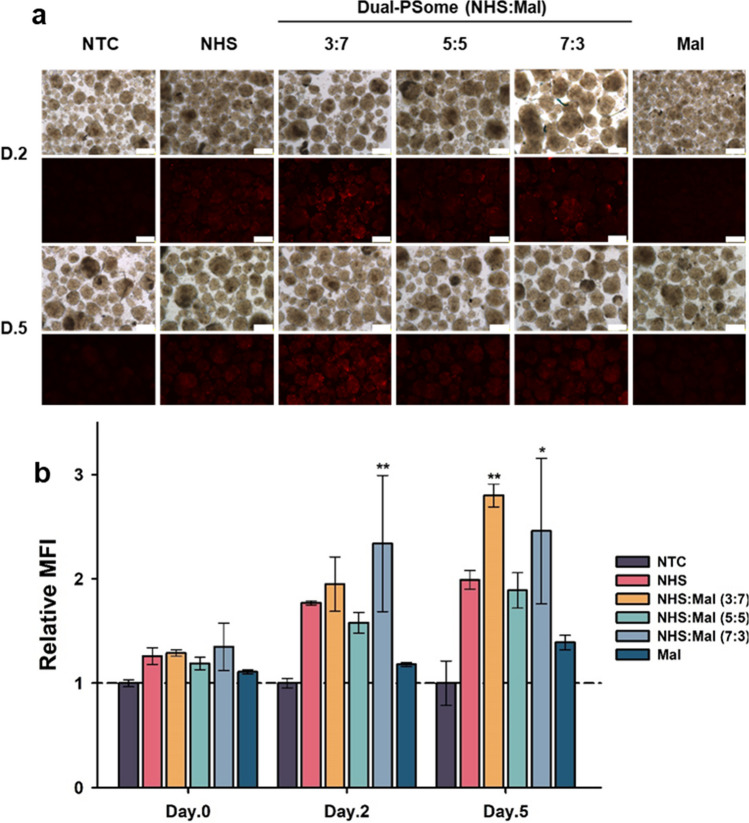
Evaluation of the nano-encapsulation efficiency on NPCCs using PSomes with diverse functional groups over a 5-day period. **a** NPCCs treated with DiI-loaded NHS, Mal, or a combination of NHS and Mal PSomes. The upper and lower rows display bright field and DiI fluorescence images, respectively. Scale bars are set to 200 μm. **b** Quantitative assessment of the MFI from DiI-loaded PSomes on nano-encapsulated NHS-, Mal-, and combined NHS and Mal-NPCCs. Sample sizes are n = 3, except for the 7:3 ratio and the nontreated control (NTC; n = 6). Data are shown as mean ± standard deviation. Statistical significances are compared with those of Mal groups

## Comparison of nano-encapsulation efficiency of PSomes with various ratios on the surface of NPCCs

It is well-known that the ECM enclosing NPCCs contains amine groups at the collagen terminals as well as specific thiol groups that may be found either inside the ECM or the overall cell membrane [19, 23]. We developed a method to use NHS and mal residues at the terminus of the encapsulation material after recognizing that they may serve as possible binding sites for encapsulation. The objective of this strategy was to selectively target and attach to amine and thiol sites. To determine the optimal ratio of NHS to Mal that would be attached to the PSomes, we performed coating experiments with PSomes using both mono and combined bifunctional groups on the NPCCs. In previous study, after a period of approximately 12 days in in vitro culture, NPCCs experienced a decline in their morphology and viability [20]. Therefore, we observed the coating efficacy for up to 5 days post nano-encapsulation on day 8 of culture, ensuring that the total culture period did not exceed 13 days. The effectiveness of nano-encapsulation, as measured by the intensity of the 1,1′-Dioctadecyl-3,3,3′,3′-tetramethylindocarbocyanine perchlorate (DiI), was collected by fluorescence microscopy and shown in Fig. 2. PSomes from all groups successfully encapsulated the surface of the NPCCs; however, it was clear that NPCCs nano-encapsulated with NHS:Mal ratios of (3:7) and (7:3) dual-PSomes demonstrated better coating efficiencies on the second and fifth days after encapsulation (Fig. 2a). In addition, validation was offered by the comprehensive quantification of the DiI intensity, which was presented as the mean fluorescence intensity (MFI). On the second and fifth days after nano-encapsulation, the NHS:Mal (3:7) and (7:3) dual-PSome nano-encapsulated NPCCs showed a considerable encapsulation advantage over the Mal-PSome group (Fig. 2b). Overall, these results highlight that NHS:Mal ratios of (3:7) and (7:3) ware considered the best configurations for dual-PSome coatings to successfully encapsulate the surface of NPCCs.

## Conformal coating of PSomes on NPCCs

**Fig. 3:**
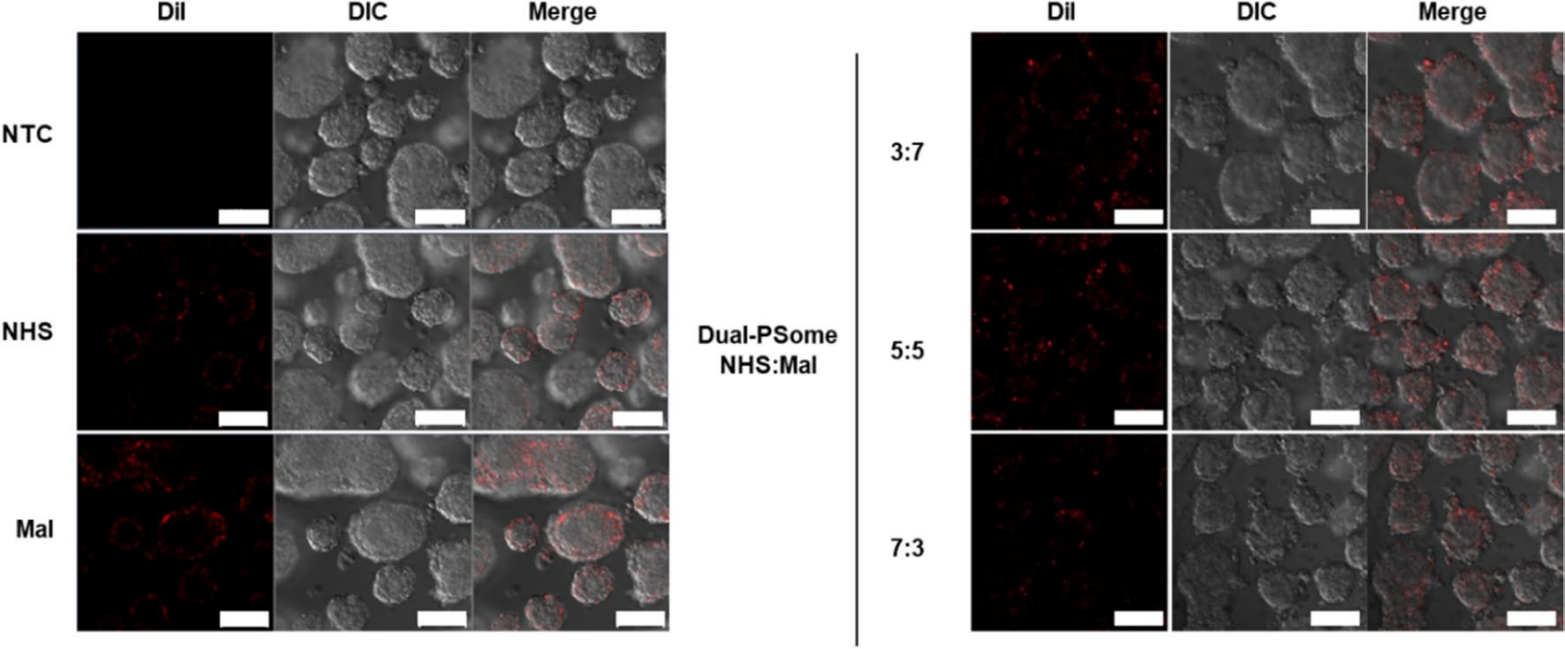
Conformal nano-encapsulation evaluation utilizing PSomes with distinct functional groups. Using a CLSM, NPCCs treated with DiI-loaded NHS-, Mal-, or a combination of NHS and Mal-PSomes were visualized after nano-encapsulation. The scale bars are set to 100 μm

Nano-encapsulation of NPCCs using PSomes has the potential benefit of lowering the transplantation volume [10]. This allows a larger amount of islets can be transplanted into the transplantation site compared to traditional encapsulation methods and this advantage may be attributed to the conformal coating on the surface of NPCCs [24]. In an attempt to provide evidence to support this notion, we used confocal laser scanning microscopy (CLSM) to examine the conformal coating after the nano-encapsulation of NPCCs with DiI-conjugated PSomes. The CLSM images clearly show that the NPCCs surfaces are conformally coated with NHS-, Mal-, and NHS:Mal dual-PSomes at ratios of (3:7), (5:5), and (7:3), respectively (Fig. 3). This result supports the theory that our nano-encapsulation method is perfectly capable of covering the surface layer of NPCCs. Owing to this accuracy, unnecessary over-coating or undesirable intracellular penetration may be avoided, which can ultimately lead to a decrease the volume of transplantation and an immunoisolation from host’s immune attack.

## Selective permeability of PSomes on NPCCs

**Fig. 4:**
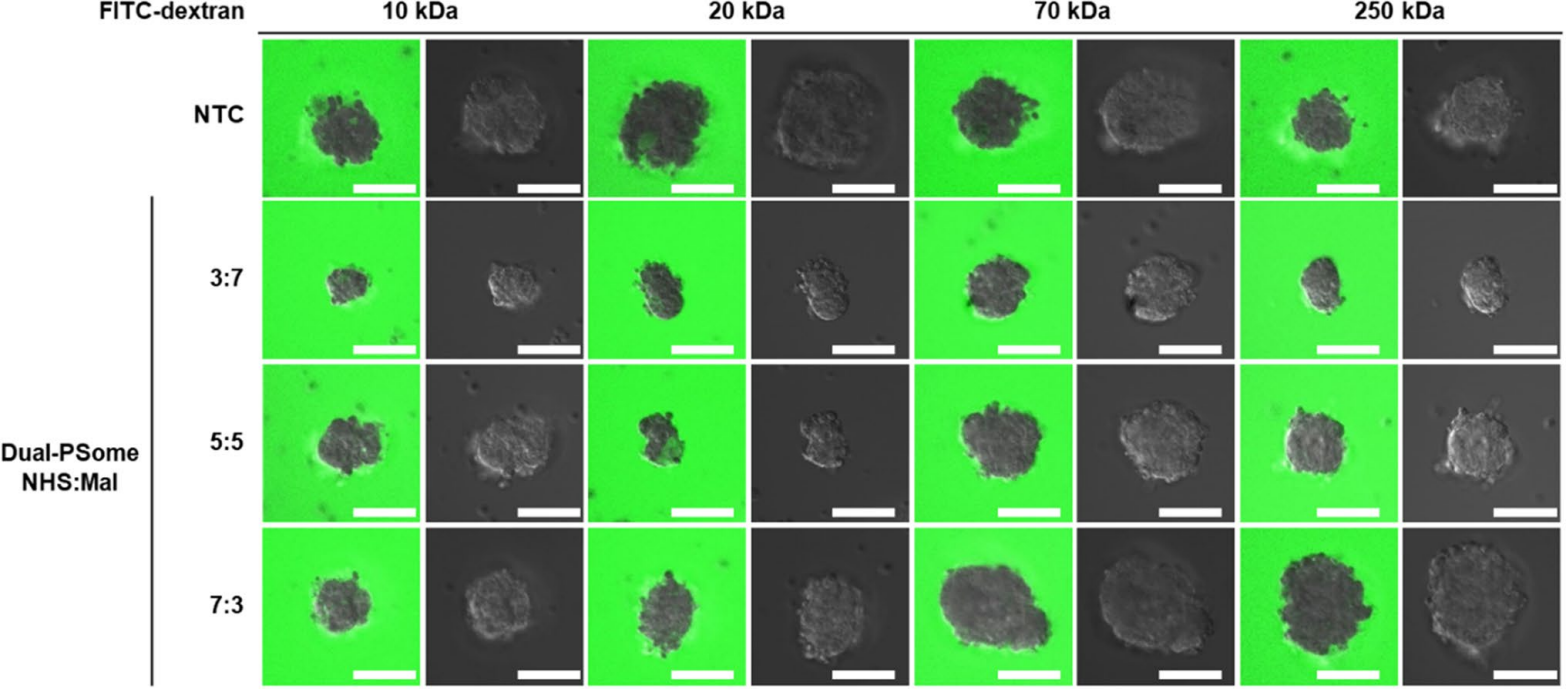
Analysis of the permeability of nano-encapsulated NPCCs with Dual-PSomes of varying functional groups. The diffusion and selectivity of dual-PSome nano-encapsulated NPCCs containing NHS and Mal (in ratios of 3:7, 5:5, and 7:3) were evaluated via a 2-h incubation with the FITC-dextran with molecular weights of 10, 20, 70, and 250 kDa. The established molecular threshold was 20 kDa. FITC fluorescence (left) and differential interference contrast (right), captured as a z-stack with CLSM. The scale bars are set to 100 μm

For cell nano-encapsulation materials, a delicate equilibrium is required; they must permit the passage of small molecules essential for NPCC survival while simultaneously blicking larger macromolecules to reduce immune reactions [25]. To examine this ability, nano-encapsulated NPCCs were exposed to fluorescein isothiocyanate (FITC)–conjugated dextrans of varying molecular weights, and their subsequent permeation into islet clusters was evaluated using CLSM. Based on the results shown in Fig. 2, we conducted selective permeability experiments using only dual-PSomes with high coating efficiency. Our findings indicated that dual-PSomes facilitate the passage of essential small molecules such as oxygen, glucose, and insulin (represented by 10- and 20-kDa FITC-conjugated dextran). While these PSomes prevented the entry of larger macromolecules, such as immune cells and antibodies (represented by 70- and 250-kDa FITC-conjugated dextran) (Fig. 4). These results suggest that Dual-PSomes are promising encapsulation materials which permits essential molecular permeation while simultaneously acting as a barrier against immune responses on NPCCs.

## T3-loaded dual-PSomes

**Fig. 5:**
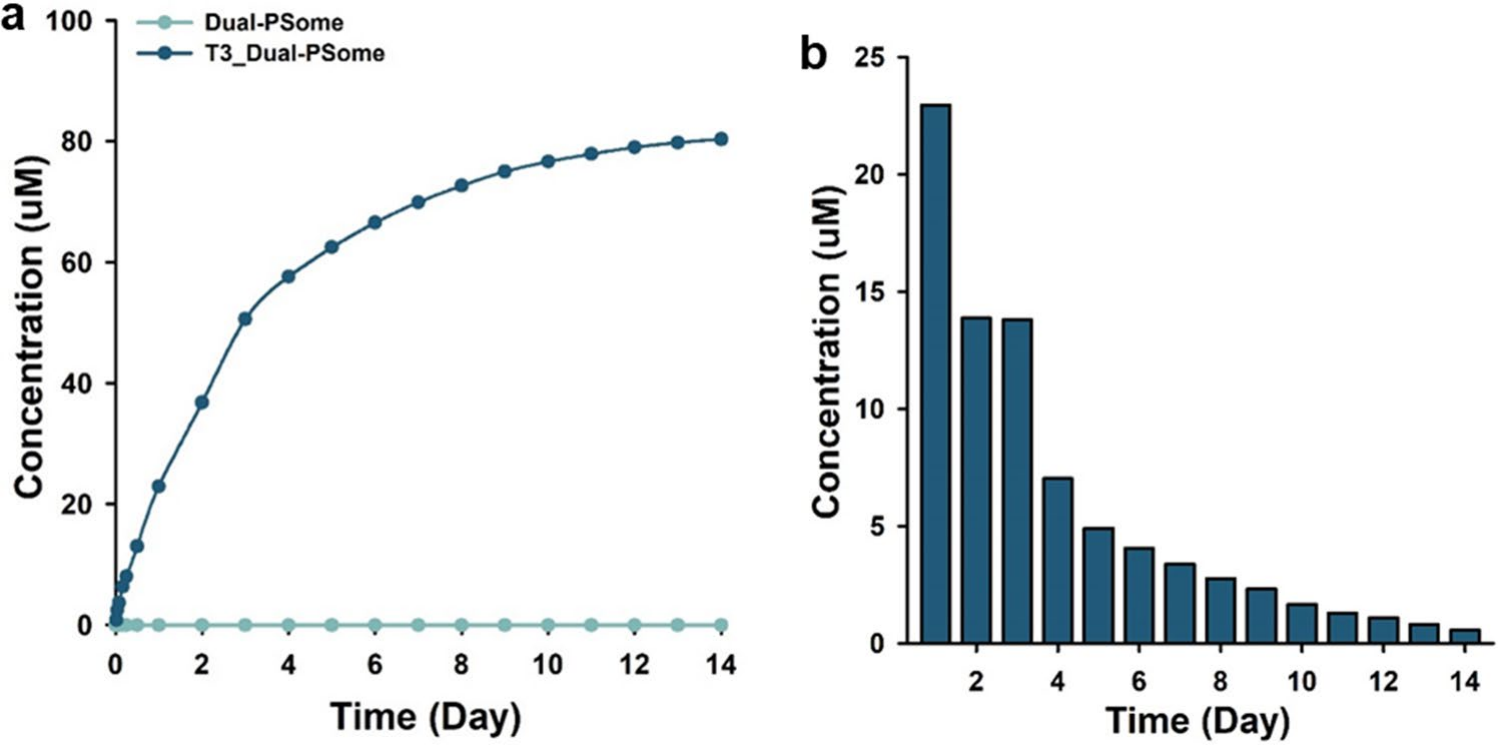
Release of T3 under control from Dual-PSomes. **a** Display of the sustained release of T3, which is encapsulated within the dual-PSomes. As a negative control, values measured from dual-PSomes without T3 present at baseline. **b** T3 release concentration against time. The graph demonstrates the concentration of the released T3 daily

One inherent difficulty associated with NPCCs is their relatively dampened response to external glucose concentrations [6, 11, 26]. This is owing to their immaturity, particularly when compared with mature APIs. Consequently, these NPCCs require a long post-transplantation period to restore normal insulin production. Therefore, extensive research has been conducted to enhance both the maturation of NPCCs and insulin secretion. T3 is an essential hormone secreted by the thyroid gland in response to the thyroid stimulating-hormone. T3 has multiple effects on cellular activities, including the enhancement of insulin secretion and islet survival in in-vitro cultures. As a result, T3 addition is frequently used to stimulate islet maturation, differentiation, and production of stem cell–derived beta cells [21, 22, 27, 28]. Based on this context, we investigated the feasibility of PSomes as a delivery carrier for the administration of T3 to NPCCs. With their amphiphilic properties, allowing for the loading of both hydrophilic and hydrophobic compounds, PSomes have emerged as promising carriers of various pharmaceuticals. The objective of our efforts to incorporate T3 into PSomes was to increase the insulin secretion capacity of beta cells within the NPCCs. Figures 3 and 4 demonstrate that dual-PSomes conformally coated the NPCCs with selective permeability. Based on the superior encapsulation efficacy observed in NHS:Mal (3:7) and (7:3) dual-PSomes, as well as the pronounced binding affinity of NHS:Mal (7:3) dual-PSome at the onset of nano-encapsulation, we chose the latter for T3 delivery to the NPCCs (Fig. 2). Our subsequent evaluation of in vitro T3 release kinetics, as measured using high-performance liquid chromatography (HPLC), yielded insightful conclusions. Quantitative analysis revealed that T3 was consistently secreted for up to 12 days after capture, with concentrations exceeding 2 µM. Surprisingly, even after 12 days, the secretion level continued to exceed 0.6 µM, resulting in the discharge of the entire entrapped T3 (Fig. 5). Notably, a 1 µM concentration of T3 is required to enhance the insulin secretion capabilities of NPCCs [22]. Although our results showed that the initial T3 release concentration from the PSome may have been excessive, that is, more than 20 µM, previous studies have reported no effect on cell viability and insulin secretory capacity up to 250 µM in rat insulin-producing cells, confirming the availability of the fabricated T3-loaded dual-PSomes [29]. Therefore, the T3-loaded dual-PSomes developed in this study are suitable agents for enhancing the performance of NPCCs. The PSomes developed for NPCC nano-encapsulation in this study efficiently loaded T3 and ensured its sustained, long-term secretion. This finding suggests an improvement in the insulin-secreting effectiveness of beta cells in NPCCs following transplantation.

## Enhanced insulin secretion capacity of NPCCs through encapsulating T3-loaded dual-PSomes

**Fig. 6:**
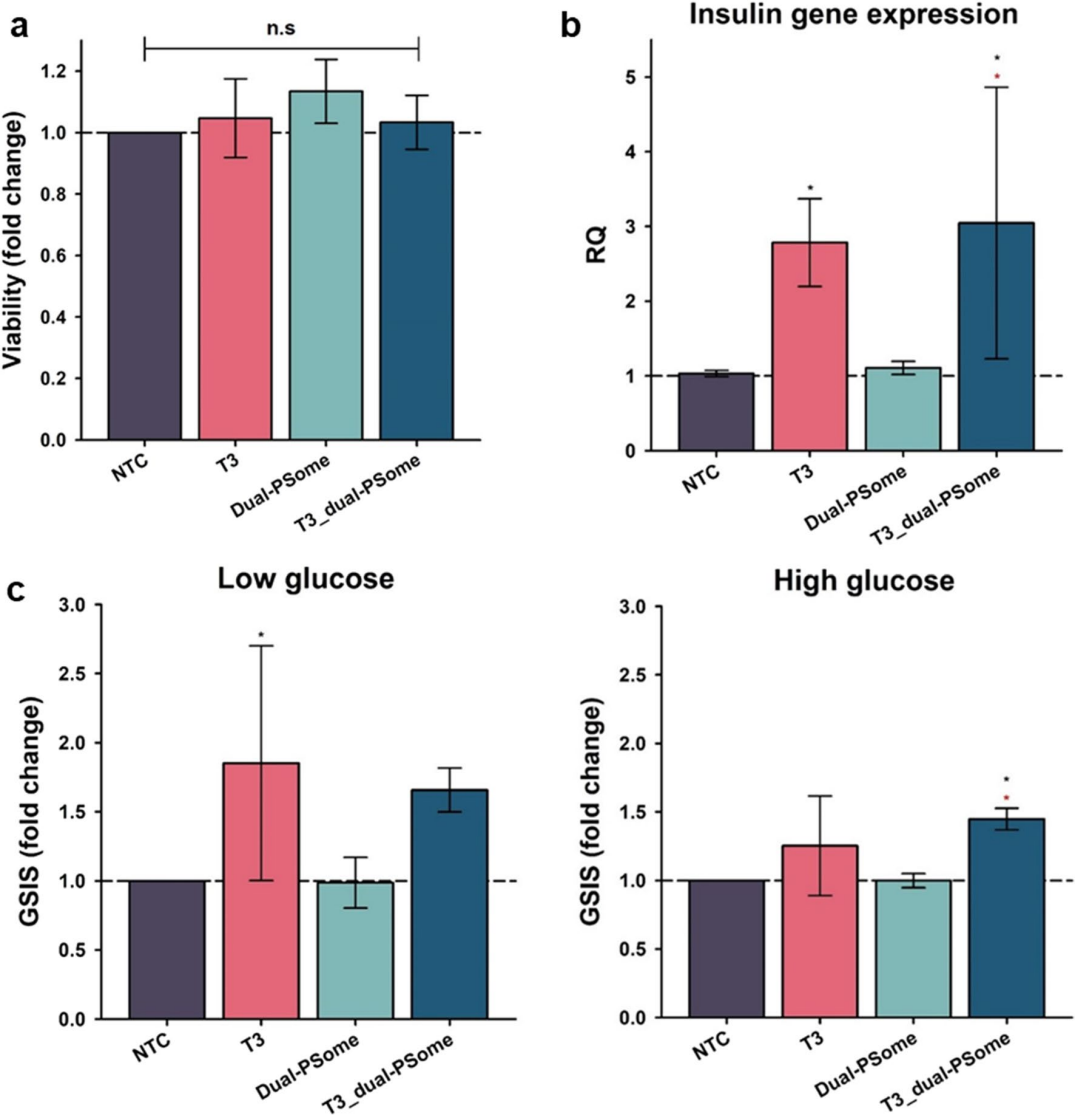
Evaluating the functionality of T3-loaded Dual-PSome nano-encapsulated NPCCs. **a** Cell viability, as determined using AO and PI staining. **b** Quantification of insulin mRNA expression using qRT-PCR. **c** Evaluation of the glucose sensitivity of NPCCs using the GSIS assay. Normalized against the NTC, all resulting data are depicted as fold changes. NPCCs were cultured without supplements (NTC), NPCCs exposed to T3 during culture (T3), NPCCs nano-encapsulated with dual-PSomes (Dual-PSome), and NPCCs nano-encapsulated with T3-loaded dual-PSomes (T3_dual-PSome) are the key groups. Sample sizes are n = 3, except for the NTC (n = 5). Data are shown as mean ± standard deviation. The symbols * are compared with NTCs and * with Dual-PSomes

The main objective of our study was to ascertain whether nano-encapsulation of T3-loaded PSomes could enhance the insulin-secretory capabilities of NPCCs. Our investigations were therefore divided into distinct groups: NPCCs encapsulated with NHS:Mal (7:3)–dual-PSome (designated as Dual-PSome) and NPCCs encapsulated with T3-loaded NHS:Mal (7:3)–dual-PSome (designated as T3_dual-PSome); NPCCs were cultured in a T3-supplemented medium (designated as T3), which served as a positive control. Arcidine orange (AO) or propidium iodide (PI) staining was used to determine the post-encapsulation viability of NPCCs. Relative quantification was normalized by nontreated control (NTC). Figure 6a shows that neither the nano-encapsulation method nor the presence of T3 compromised the viability of NPCCs. As a supplementary measure, we investigated whether PSomes affect the viability of other standard cell lines. Nano-encapsulation of HeLa cells with PSomes revealed no discernible effects on cell viability, further confirming the safety of PSomes (Details in Supplement fig. S1). A significant increase in insulin gene expression (measured using quantitative real-time polymerase chain reaction [qRT-PCR]) between the T3 and T3_dual-PSome groups and their NTC and Dual-PSome counterparts was a crucial finding. The observed equality in insulin gene expression between T3 and T3_dual-PSomes was intriguing, suggesting that T3_dual-PSomes may mimic the functional properties of T3 presence in the culture medium (Fig. 6b). Expanding our scope, we conducted a glucose-stimulated insulin secretion (GSIS) assay to determine whether enhanced insulin secretory propensity corresponds to an increased response to exogenous glucose concentrations. Figure 6c demonstrates that, consistent with our previous findings, NPCCs nano-encapsulated with T3_ dual-PSomes secreted more insulin than NTC and Dual-PSomes. Our findings demonstrated that T3_dual-PSomes have the potential to enhance the functional capabilities of NPCCs. Also, this was accomplished by efficiently retaining and releasing T3 while preserving the innate vitality of NPCCs.

## Conclusion

In this study, dual-PSomes conjugated with NHS and Mal were used to nanoencapsulate NPCCs, thereby augmenting their binding capacity via a double covalent bond. The potential of NPCCs obtained from neonatal piglets to treat T1D has become increasingly evident. However, the problem of transplant rejection caused by xenoantigens in NPCCs remains a significant obstacle. The dual-PSomes, which were utilized to demonstrate a novel nano-encapsulation technique, were attached to NPCCs via a dual covalent bond, thereby enhancing the encapsulation efficiency and shielding the xenoantigens. The benefits of T3 encapsulation in enhancing the glucose sensitivity of NPCCs were demonstrated, and the optimal ratio of NHS to Mal for this purpose was determined. Our nanoplatform not only increased the ability of NPCCs to secrete insulin but also promoted insulin gene expression. By providing an effective immunoisolation barrier for NPCCs and resolving their functional immaturity, the proposed dual-targeting strategy has the potential to fundamentally modify the field of islet transplantation. Utilizing the unique properties of T3 and dual PSomes, our research paves the way for future therapeutic approaches that are more likely to be successful in the treatment of T1D, leading to innovative advances in the field of islet xeno-transplantation.

### Supplementary Information

Additional file1 (DOCX 123 KB)

## Data Availability

The datasets generated during and/or analysed during the current study are available from the corresponding author on reasonable request.
