# Supplementary material for: Dual-targeted nano-encapsulation of neonatal porcine islet-like cell clusters with triiodothyronine-loaded bifunctional polymersomes
Source: Discov Nano. 2024 Feb 5;19(1):23. doi: 10.1186/s11671-024-03964-3 (PMC10844179; doi:10.1186/s11671-024-03964-3)
Supplement: Supplementary file 1 — Additional file1 (DOCX 123 KB) [file 11671_2024_3964_MOESM1_ESM.docx]

**Supporting Information for**

**Dual-Targeted Nano-Encapsulation of Neonatal Porcine Islet-Like Cell Clusters with Triiodothyronine-Loaded Bifunctional Polymersomes**

**Sang Hoon Lee^1,2*^, Minse Kim^2,3*^, Eun Jin Lee^1^, Sun Mi Ahn^1^, Yu-Rim Ahn^2,3^, Jaewon Choi^2,3^, Jung-Taek Kang^1†^, and Hyun-Ouk Kim^2, 3†^**

^1^MGENSolutions Biotechnology Research Institute, Seoul, 06688, Republic of Korea.

^2^Department of Biotechnology and Bioengineering, Kangwon National University, Chuncheon, Gangwon-do, 24341, Republic of Korea

^3^Biohealth-machinery Convergence Engineering, Kangwon National University, Chuncheon, Gangwon-do, 24341, Republic of Korea

* These authors contributed equally.

† Corresponding author: Prof. Hyun-Ouk Kim, Department of Biotechnology and Bioengineering, Kangwon National University, Chuncheon, Gangwon-do, 24341, Republic of Korea

**Supplementary Table 1.** Yield, viability, and functionality of NPCCs 6 days after isolation

| # | Body weight (Kg) | Pancreas weight (g) | NPCCs Yield  (IEQ/gram, pancreas weight) | NPCCs Yield  (IEQ/pancreas) | Viability (%) | ※Low (pmole/L  /pg DNA/h) | ※High (pmole/L/  pg DNA/h) |
| --- | --- | --- | --- | --- | --- | --- | --- |
| 1 | 1.2 | 2.2 | 9191.0 | 20,221.0 | 83.9 | 3.7 | 6.8 |
| 2 | 1.5 | 2.3 | 21,469.0 | 49,380.0 | 87.3 | 2.8 | 5.3 |
| 3 | 1.5 | 2.7 | 22,563.0 | 60,920.0 | 83.7 | 7.6 | 9.3 |
| 4 | 1.2 | 1.7 | 27,832.0 | 47,314.0 | 88.9 | 6.6 | 9.3 |
| 5 | 1.1 | 1.4 | 24,644.0 | 34,501.0 | 94.0 | 7.5 | 11.2 |
| Aver | 1.3 | 2.1 | 21,139.8 | 42,467.2 | 87.6 | 5.6 | 8.4 |
| SD | 0.2 | 0.5 | 6355.7 | 13,929.4 | 3.8 | 2.0 | 2.1 |

- Low and high values represent the insulin quantities obtained from the glucose-stimulated insulin secretion assay. SD, standard deviation; IEQ; islet equivalent; NPCCs, neonatal porcine islet-like cell clusters.


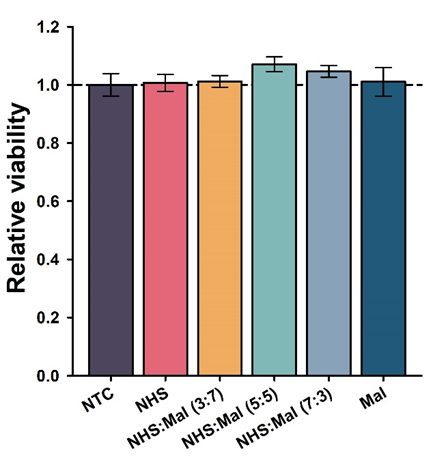


**Supplement fig 1.** Viability of HeLa human cell lines in response to polymersomes (PSomes). The viability of the PSome nano-encapsulated HeLa cells is measured using the MTT assay (n=8).


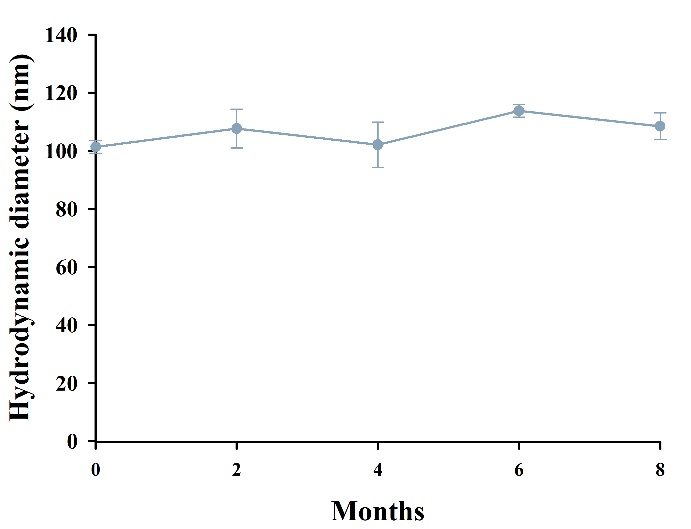


**Supplement fig 2.** The NHS-Mal (7:3)-Polymersome (Dual-PSome) stability was assessed using DLS for up to 8 months.
